# Metal-chloroquine derivatives as possible anti-malarial drugs: evaluation of anti-malarial activity and mode of action

**DOI:** 10.1186/1475-2875-13-471

**Published:** 2014-12-03

**Authors:** Maribel Navarro, William Castro, Marilyn Madamet, Rémy Amalvict, Nicolas Benoit, Bruno Pradines

**Affiliations:** Centro de Química, Instituto Venezolano de Investigaciones Científicas (IVIC), Caracas, Venezuela; Directoria de Metrologia Aplicada á ciências da vida, Instituto Nacional de Metrologia, Normalização e Qualidade Industrial, Rio de Janeiro, Brazil; Equipe Résidente de Recherche en Infectiologie Tropicale, Institut de Recherche Biomédicale des Armées, Hôpital d’Instruction des Armées Laveran, Marseille, France; Aix Marseille Université, Unité de Recherche sur les Maladies Infectieuses et Tropicales Emergentes, UM 63, CNRS 7278, IRD 198, Inserm 1095, Marseille, France; Centre National de Référence du Paludisme, Marseille, France; Unité de Parasitologie et d’Entomologie, Département des Maladies Infectieuses, Institut de Recherche Biomédicale des Armées, Brétigny sur Orge, France

**Keywords:** Malaria, *Plasmodium falciparum*, Anti-malarial, *in vitro*, Resistance, Gold, Platinum, Chloroquine, β-hematin

## Abstract

**Background:**

Malaria still has significant impacts on the world; particularly in Africa, South America and Asia where spread over several millions of people and is one of the major causes of death. When chloroquine diphosphate (CQDP) lost its efficiency as a first-line anti-malarial drug, this was a major setback in the effective control of malaria. Currently, malaria is treated with a combination of two or more drugs with different modes of action to provide an adequate cure rate and delay the development of resistance. Clearly, a new effective and non-toxic anti-malarial drug is urgently needed.

**Methods:**

All metal-chloroquine (CQ) and metal-CQDP complexes were synthesized under N_2_ using Schlenk techniques. Their interactions with haematin and the inhibition of β-haematin formation were examined, in both aqueous medium and near water/n-octanol interfaces at pH 5. The anti-malarial activities of these metal- CQ and metal-CQDP complexes were evaluated *in vitro* against two strains, the CQ-susceptible strain (CQS) 3D7 and the CQ-resistant strain (CQR) W2.

**Results:**

The previously synthesized Au(CQ)(Cl) (1), Au(CQ)(TaTg) (2), Pt(CQDP)_2_Cl_2_ (3), Pt(CQDP)_2_I_2_ (4), Pd(CQ)_2_Cl_2_ (5) and the new one Pd(CQDP)_2_I_2_ (6) showed better anti-malarial activity than CQ, against the CQS strain; moreover, complexes 2, 3 and 4 were very active against CQR strain. These complexes (1–6) interacted with haem and inhibited β-haematin formation both in aqueous medium and near water/n-octanol interfaces at pH 5 to a greater extent than chloroquine diphosphate (CQDP) and other known metal-based anti-malarial agents.

**Conclusions:**

The high anti-malarial activity displayed for these metal-CQ and metal-CQDP complexes (1–6) could be attributable to their effective interaction with haem and the inhibition of β-haematin formation in both aqueous medium and near water/n-octanol interfaces at pH 5.

**Electronic supplementary material:**

The online version of this article (doi:10.1186/1475-2875-13-471) contains supplementary material, which is available to authorized users.

## Background

Approximately 3.3 billion people, one half of the world’s population, live in at-risk regions for malaria infection. In 2013, an estimated 207 million episodes of malaria occurred, and approximately 627,000 people died [1]. Of the five typically recognized *Plasmodium* species causing this disease in humans, *Plasmodium falciparum* is responsible for about 95% of malaria worldwide and has a mortality rate of 1–3%, and *Plasmodium vivax* for most morbidity, additionally representing a reservoir of latent infection that hampers current control and future elimination efforts [2, 3].

When chloroquine resistant (CQR) malaria parasites started to emerge in Asia and South America and spread from Asia to Africa, and lost its efficiency as a first line anti-malarial drug, this was a major setback to the effective control of malaria. Currently, malaria is treated with a combination of two or more drugs with different modes of action to provide an adequate cure rate and delay development of resistance.

A strategy for the development of alternative therapies against malaria has been based on the modification of compounds with known or potential activity through the incorporation of a transition metal into the molecular structure, this modification is important within biological systems due to the binding capability and reactivity of the transition metals, which are determined by the *d* orbitals [4]. A variety of metal complexes has been developed as possible anti-malarial agents [5–8], progressed from simple “synthesis/activity” to complex insights into their mechanisms of action, these concepts open new perspectives in drug design, for example Sanchez-Delgado and Navarro groups have proposed the modification of CQ through the incorporation of a transition metal into the molecular structure. Indeed, several metal centres (Rh, Ru, Ir and Au) have been used finding that the most significant results have been obtained for the centers of ruthenium [9, 10] and gold [11, 12].

Encouraged by these interesting biological results the studies of the mechanism of action of these metal-chloroquine complexes ([RuCl_2_(CQ)]_2_ and [Au(CQ)(PPh_3_)]PF_6_) were evaluated on two important targets, Fe(III)PPIX and DNA [13, 14], finding that the main mechanism of anti-malarial action of these [RuCl_2_(CQ)]_2_ and [Au(CQ)(PPh_3_)]PF_6_ complexes against resistant strains of *P. falciparum* was the inhibition of β-haematin formation. Both the enhanced activity and the ability of these compounds to lower CQ resistance are related to the high lipophilicity of the metal complexes and the important structural modification of the CQ structure imposed by the presence of the metal-containing fragment.

Taking into account the importance of the interactions of these metal compounds with the specific targets Fe(III)PPIX and the relevance of the inhibition of β-haematinin order to increase anti-plasmodial activity and avoid parasite resistance, the interaction of six metal-CQ (metal: Au, Pt and Pd) complexes with ferriprotoporphyrin, their ability to inhibit the haem aggregation at water/lipid interfaces and their anti-malarial activity against 3D7 and W2 strains (CQ-susceptible strain and CQ-resistant strain, respectively) were assessed in the present work. Worthy of mentioning, that the cytotoxic activity against the six tumor cell lines, of five of these metal-CQ complexes have already been reported [15, 16] that significant activity was attributed mainly to their interaction with the DNA. Additionally, the synthesis and characterization of the new Pd(CQDP)_2_I_2_ complex were reported here.

## Methods

All manipulations were carried out under N_2_ using common Schlenk techniques. Solvents were purified by standard procedures immediately prior to use. Chloroquine diphosphate (CQDP), chloride haemin, buffers and solvents were purchased from Sigma-Aldrich. All other commercial reagents were used without further purification. Metal–CQ and Metal–CQDP (1–5) complexes were prepared according to the literature [15–17]. The NMR spectra were obtained in a DMSO solution in a Bruker AVANCE 500 spectrometer. ^1^H NMR shifts were recorded relative to residual proton resonances in the deuterated solvent. IR spectra were obtained with a Nicolet Magna IR 560 spectrometer. ESI mass spectra were obtained by using a Thermo FINNIGAN LXQ with methanol as the solvent. UV–vis spectra and all the spectrophotometric studies experiments were performed on an Agilent 8453 diode-array spectrophotometer.

### Synthesis and characterization of complex Pd(CQDP)_2_I_2_ (6)

A solution of K_2_[PdCl_4_] (65.3 mg, 0.2 mmol) in water (20 mL) was stirred until complete dissolution was achieved, an excess (20-fold) of KI was added. Then, CQDP dissolved in water (206.4 mg, 0.4 mmol) was added. The stirring was continued for 1 h at room temperature, and a brown precipitate was obtained. This was collected by filtration, washed with water, and dried under vacuum. Spectroscopic and analytical characterization of this complex was described in Additional file 1.

### *In vitro*anti-malarial activity

The two strains, 3D7, the CQ-susceptible strain (isolated in West Africa; obtained from MR4, VA, USA), and W2 (isolated in Indochina; obtained from MR4, VA, USA), the CQ-resistant strain, were maintained in culture in RPMI 1640 (Invitrogen, Paisley, UK), supplemented with 10% human serum (Abcys S.A. Paris, France) and buffered with 25 mM HEPES and 25 mM NaHCO_3_. Parasites were grown in A-positive human blood (Etablissement Français du Sang, Marseille, France) under controlled atmospheric conditions that consisted of 10% O_2_, 5% CO_2_ and 85% N_2_ at 37°C with a humidity of 95%.

The two strains were synchronized twice with sorbitol before use [18], and clonality was verified every 15 days through PCR genotyping of the polymorphic genetic markers *msp1* and *msp2* and microsatellite loci [19, 20]; additionally, clonality was verified each year by an independent laboratory from the Worldwide Anti-malarial Resistance Network (WWARN).

Chloroquine diphosphate (CQDP) was purchased from Sigma (Saint Louis, MO). CQDP was resuspended in water in concentrations ranging between 5 to 3200 nM. The synthetic compounds were resuspended in DMSO and then diluted in RPMI-DMSO (99v/1v) to obtain final concentrations ranging from 1 nM to 100 μM.

For *in vitro* isotopic microtests, 25 μL/well of anti-malarial drug and 200 μL/well of the parasitized red blood cell suspension (final parasitaemia, 0.5%; final haematocrit, 1.5%) were distributed into 96 well plates. Parasite growth was assessed by adding 1 μCi of tritiated hypoxanthine with a specific activity of 14.1 Ci/mmol (Perkin-Elmer, Courtaboeuf, France) to each well at time zero. The plates were then incubated for 48 h in controlled atmospheric conditions. Immediately after incubation, the plates were frozen and thawed to lyse erythrocytes. The contents of each well were collected on standard filter microplates (Unifilter GF/B; Perkin-Elmer) and washed using a cell harvester (Filter-Mate Cell Harvester; Perkin-Elmer). Filter microplates were dried, and 25 μL of scintillation cocktail (Microscint O; Perkin-Elmer) was placed in each well. Radioactivity incorporated by the parasites was measured with a scintillation counter (Top Count; Perkin-Elmer).

The IC_50_, the drug concentration able to inhibit 50% of parasite growth, was assessed by identifying the drug concentration corresponding to 50% of the uptake of tritiated hypoxanthine by the parasite in the drug-free control wells. The IC_50_ value was determined by non-linear regression analysis of log-based dose–response curves (Riasmart™, Packard, Meriden, USA). IC_50_ are expressed as geometric means of 6 experiments.

### Interaction with haematin

The association constant of compounds metal-CQ with ferriprotoporphyrin IX (Fe(III)PPIX) was measured as described previously [21]. Briefly, a stock solution of haemin was prepared by dissolving 3.5 mg of haemin in 15 mL DMSO. Aqueous-DMSO (40% v/v) solutions of Fe(III)PPIX (pH 7.5) were prepared daily by mixing 140 μL haemin stock solution with 4 mL DMSO and 1 mL 0.2 M (Tris; Tris(hydroxymethyl)aminomethane) Tris buffer (pH 7.5) and completed to 10 mL with doubly distilled deionized water. The concentration of Fe(III)PPIX in this solutions was 4 μM and absorbance readings were recorded at 402 nm. The reference cell containing 40% v/v DMSO, 0.020 M Tris pH 7.5 was also titrated with each complex in order to blank out the absorbance of the drug. The data were fitted to the equation A = (A_0_ + A_∞_K[C])/(1 + K[C]) for a 1:1 complexation model using nonlinear least squares fitting, strictly following the procedure of Egan *et al.* [22], with omission of the first few data points at very low drug-to-haematin ratio, where the fit is poor. A_0_ is the absorbance of haemin before addition of complex, A_∞_ is the absorbance for the drug–haemin adduct at saturation, A is the absorbance at each point of the titration, and K is the conditional association constant.

### Inhibition of β-haematin formation

The transformation of haemin to β-haematin in acidic acetate solutions was studied using infrared spectroscopy using the method developed by Egan *et al.* [22]. The IC_50_ of β-Haematin formation in buffer assay was performed according to Dominguez [23]. Briefly, a solution of haemin (50 μL, 4 mM), dissolved in DMSO, was distributed in 96-well micro plates. The complex was dissolved in DMSO and added in triplicate in test wells (50 μL) to final concentrations of 0–20 mM/well. Controls contained water and DMSO. Haemozoin formation was initiated by the addition of acetate buffer (100 μL 0.2 M, pH 4.4). Plates were incubated at 37°C for 48 h to allow completion of the reaction and centrifuged at 4,000 RPM × 15 min. After discarding the supernatant, the pellet was washed twice with DMSO (200 μL) and finally dissolved in NaOH (200 μL, 0.2 N). The solutions were further diluted 1:2 with NaOH (0.1 N) and absorbance recorded at 405 nm. The results were expressed as a percentage of inhibition of haemozoin formation.

β-Haematin formation at a water/octanol interface was followed according to a method proposed by Egan and coworkers [24] and modified by Martínez *et al.* [25]. Haemin was dissolved in 0.1 M NaOH solution to generate haematin and acetone was added until the acetone: water ratio was 4: 6; the final solution contained 15 mg haematin/mL. A sample of this solution (200 μL) was carefully introduced close to the interface between n-octanol (2 mL) and aqueous acetate buffer (5 mL, 8 M; pH 4.9) in a cylindrical vial with an internal diameter of 2.5 cm. The mixture was incubated at 37°C for 2 h and at the end of the incubation the product (β-haematin) was isolated by centrifugation. The pellet was collected and washed twice with DMSO (2 mL), centrifuged again for 20 min, washed with 2 mL of ethanol and finally dissolved in 25 mL of 0.1 M NaOH for spectrophotometric quantification. For the haem aggregation inhibition activity measurements the appropriate amount of the drug (23 mM in DMSO) was dissolved; after stirring for 30 min to equilibrate the drug between the two phases, the haematin solution was added close to the interface and the procedure was followed as described above. All experiments were performed in triplicate.

## Results and discussion

### Synthesis and characterization of complex Pd(CQDP)_2_(I)_2_ (6)

**Figure 1:**
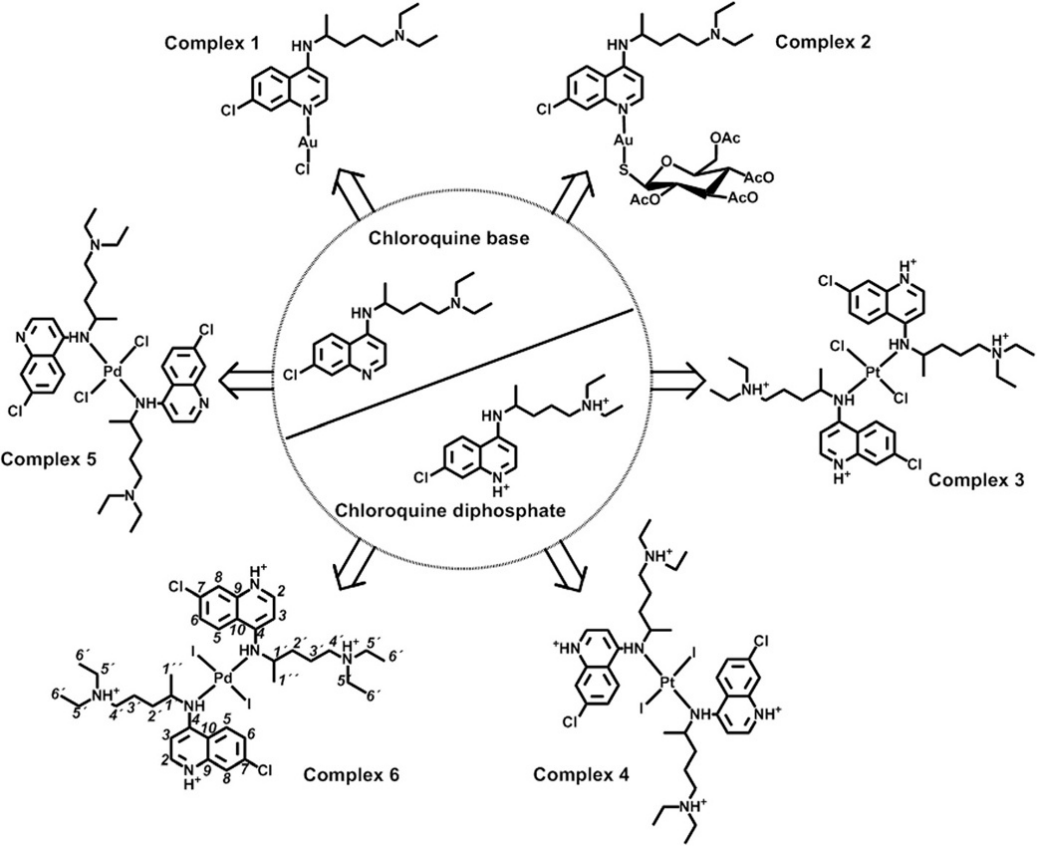
**Structure of metal-chloroquine and metal chloroquine diphosphate evaluated.**

The palladium-chloroquine diphosphate complex was synthesized at room temperature in water. An excess of KI was added to the solution of K_2_[PdCl_4_] in order to displace all chloride ligands; subsequently, CQDP was added in a ratio 2:1 with respect to palladium salt, displaced two iodide ligands leads to the new complex(6), which was isolated in good yields as air stable brown solid. Elemental analyses of this complex are in agreement with the molecular formula proposed. The IR spectra of the complexes displayed peaks clearly associated with the presence of the coordinated CQDP. The ESI-MS spectrum of complex displayed a peak of high intensity corresponding to a molecular ion (M - 4H_3_PO_4_) at m/z 1000.03. The molar conductivity values obtained for the complex is in the range for 1:4 electrolytes dissolved in DMF [26], corresponding to four phosphates (H_2_PO_4_^−^) of CQDP in the complex. All NMR signals could be unequivocally assigned on the basis of 1D and 2D, Correlation spectroscopy (COSY), Heteronuclear Multiple Quantum Correlation (HMQC) and Heteronuclear Multiple Bond Correlation (HMBC) experiments for both complexes (for complete NMR data see Annex 1; atom numbering for CQDP in Figure 1). The ^1^H and ^31^C chemical shift variation of each signal with respect to those of the free ligand (Δδ) was used as a parameter to deduce the mode of bonding of CQDP to the metal. It has been previously shown [9–12, 27] that the largest variations are always observed for the protons and carbons located in the vicinity of the N-atom attached to the metal. The largest shift with respect to the free ligand (CQDP) was observed for NH and H1’ in the ^1^H NMR spectra and C4 in the ^31^C NMR spectra (Table 1). All other chloroquine protons and carbons showed smaller displacements, indicating that CQDP is bound to the palladium through the NH atom of the secondary amine, a good donor site in this molecule. Additionally, one signal was observed in the ^31^P-NMR corresponding to the H_2_PO_4_^−^ group of CQDP (see Additional file 1). Based on the experimental data available, the formulation for the new palladium-chloroquine diphosphate compounds corresponds to 16-electron Pd(II) complexes in the usual d^8^ square planar coordination geometry, of *trans* configuration due to steric repulsion between the two chloroquine diphosphate ligands.

**Table 1:** **Displacement of protons and carbons (Δδ, ppm) of the CQDP in complex 6 with respect to the free ligands (DMSO as solvent)**

Protons	δ (ppm)	Δδ (ppm)	Carbons	δ (ppm)	Δδ (ppm)
H6'	1.16	0.16	C9	155.38	3.26
H1''	1.31	0.06	C2	143.62	2.35
H2' and H3'	1.72	0.09	C4	138.99	*9.72*
H4' and H5'	3.10	0.03	C7	138.66	3.08
H1'	4.16	*0.31*	C6	127.29	2.71
H3	6.99	0.13	C5	126.25	1.06
H6	7.82	0.21	C8	119.59	0.28
H8	7.91	0.11	C10	115.85	2.00
H2	8.58	0.04	C3	99.31	0.02
H5	8.64	0.15	C4'	51.09	0.32
NH	8.86	*1.80*	C1'	49.67	1.55
NH^+^	13.65	-	C2'	32.44	0.68
			C3'	20.62	0.83
			C1''	20.00	0.26
			C6’	9.11	0.51

Since the biological test are carried out using DMSO solution, it is important to mention that the metal complexes reported here are stable in this solvent, their NMR spectra in DMSO-d_6_ remain unchanged for several days at room temperature, showing no evidence of displacement of the CQ ligand by the solvent or any decomposition of these complexes. Worthy of mentioning is that only AuClCQ showed color change after 12 hours, however the ^1^HNMR spectrum showed the characteristic peaks for CQ ligand coordinated to the gold.

### Anti-malarial activity

*In vitro* anti-malarial activity studies were carried out in *Plasmodium falciparum* parasite, using a chloroquine-susceptible (CQS) strain (3D7) and chloroquine-resistant (CQR) strain (W2); the results are given in Table 2. The anti-malarial drug CQDP was used as a reference. All tested metal-CQ and metal-CQDP complexes displayed very high anti-malarial activity against the CQS strain (3D7), in general all metal complexes showed better activity than CQDP, except Pd(CQDP)_2_I_2_ which showed the less activity (IC_50_ = 24 nM). Suggesting that, metals like gold and platinum achieved to improve the antiplasmodial activity of CQDP. However, this trend was not observed when the studied metal complexes were tested against the CQR strain (W2), the result revealed that Pt(CQDP)_2_Cl_2_ was the most active compound (IC_50_ = 89 nM) of these series, followed by Au(CQ)(TaTg) and Pt(CQDP)_2_I_2_ which displayed similar antiplasmodial activity IC_50_ = 175 nM and IC_50_ = 177 nM respectively; these three complexes were clearly more active that CQDP (IC_50_ = 406 nM), the rest of the studied metal complexes were less active than CQDP, although not clear trend was observed, it is noticeable that Pt(CQDP)_2_Cl_2_ was the most active complex against CQS and CQR. The IC_50_ values found in the 3D7 CQS strain are equivalent to those found in CQS laboratory strains or field isolates for the ferroquine, the 7-chloro-4-[(2-N,N-dimethylaminomethyl)ferrocenyl-methylamino]quinoline, ruthenoquine or 2-phenylindoles and 3-ferrocenylmethyl-2-phenylindoles but those obtained in the W2 CQR strain are higher than those found in CQR laboratory strains or field isolates for the two metalloquines [28–32]. However, the *in vitro* activity of these different compounds exhibited higher activity than those in the micromolar range obtained with pyrazole palladium or platinum complexes [33], rhenium-4-aminoquinolines [34], ferrocene-ciprofloxacin complexes [35, 36] and ferrocenyl-chalcones [37]. All tested metal complexes are more active against CQS than CQR. These data suggest that the activity of present compounds is correlated with that of CQ. Previous studies suggested that metal coordination to CQ reduces or abolished cross-resistance [28, 31]. The ferroquine and ruthenoquine were correlated to each other but not with CQ, confirming the lack of cross-resistance. However, in some works, some rhenium bioorganometallics based on the 4-aminoquinoline structure were less active *in vitro* against the W2 CQR strain than the 3D7 CQS [34]. A larger number of strains with several susceptibility profiles should be necessary to properly assess the cross-resistance between the present compounds and CQ and conclude.

**Table 2:** ***In vitro*** **antimalarial activity and interaction with hemin, inhibition of β-hematin formation**

Compound	Interaction with hemin	Inhibition of β-hematin formation				
	3D7(nM) ^a^	W2(nM) ^a^	Log K ^d^	IR	HAI _50_ (mM) in buffer ^e^	HAI _50_ (mM) in interface ^f^
(**1**) Au(CQ)(Cl)	10 (1.80)	483 (0.84)	4.76 ± 0.20	+	0.82 ± 0.04 (0.2)	0.99 ± 0.08 (2.3)
(**2**) Au(CQ)(TaTg)	15 (1.20)	175 (2.32)	4.63 ± 0.03	+	0.75 ± 0.05 (0.2)	2.32 ± 0.11 (0.9)
(**3**) Pt(CQDP)_2_(Cl)_2_	7 (2.57)	89 (4.56)	4.89 ± 0.10	+	1.88 ± 0.11 (0.1)	2.09 ± 0.61 (1.1)
(**4**) Pt(CQDP)_2_(I)_2_	10 (1.80)	177 (2.29)	4.85 ± 0.09	+	0.52 ± 0.06 (0.3)	1.86 ± 0.06 (1.2)
(**5**) Pd(CQ)_2_(Cl)_2_	12 (1.50)	653 (0.62)	4.09 ± 0.04	+	0.46 ± 0.02 (0.3)	1.88 ± 0.81 (1.2)
(**6**) Pd(CQDP)_2_(I)_2_	24 (0.75)	608 (0.67)	4.45 ± 0.02	+	0.34 ± 0.08 (0.4)	2.21 ± 0.12 (1.0)
CQDP	18 (−−)	406 (−−)	5.01 ± 0.01	+	0.15 ± 0.03 (1)	2.23 ± 0.09 (1.0)

HAI_50_ is the drug-to-hemin ratio required to inhibit 50% of heme aggregation against a control experiment in the absence of drugs.

^a^Values in parentheses are the relative activity with respect to CQDP. The IC_50_ values are the mean of 6 experiments.

^d^pH ~ 5.

^e^After 24 h reaction.

^f^After 2 h reaction.

### Interaction with haemin and inhibition of β-haematin formation

**Figure 2:**
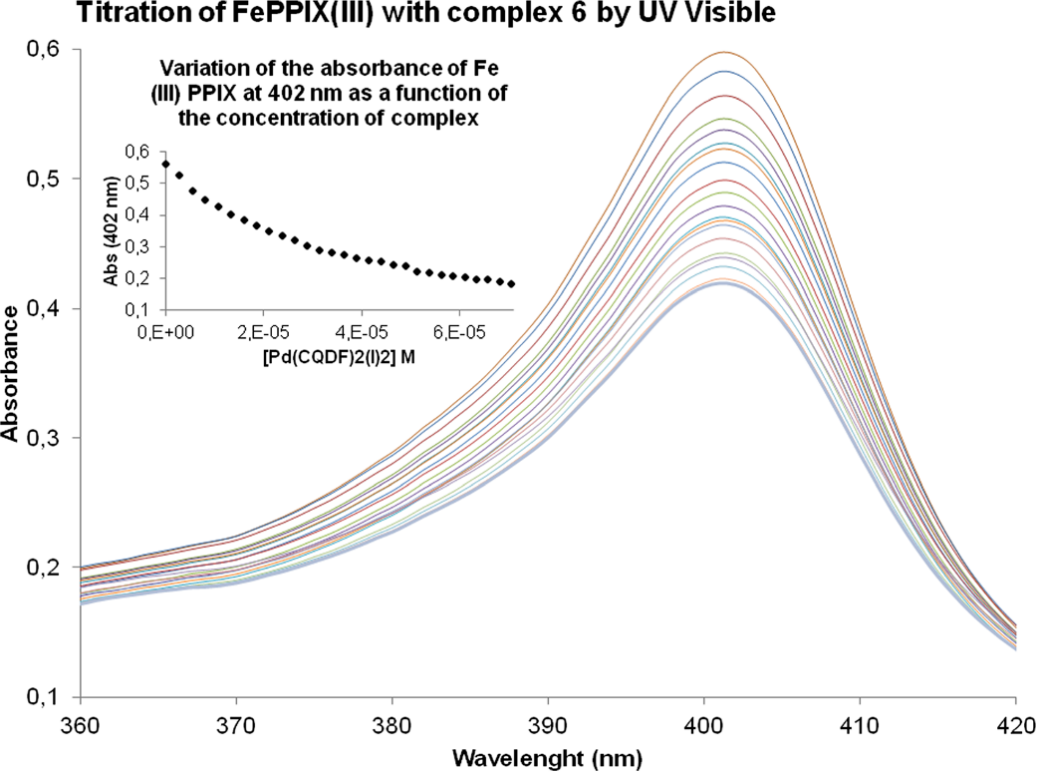
**Variation in absorbance of Fe(III)PPIX at 402 nm as a function of complex 6 concentration.** Conditions: 40% DMSO, apparent pH 7.5, 0.020 M HEPES buffer, [Complex 6] = 26.98x10^−6^ M 25°C. Insert: log [(A-A_o_) (A_∞_-A)] vs log [complex 6] (M).

Chloroquine and its metal derivatives have shown that they can act through the formation of adducts with ferriprotoporphyrin IX, thus blocking haemozoin formation [15, 16, 38]. Indeed, in previous studies we have indicated that the anti-malarial activity of metal-CQ complexes often correlated with the interaction with haemin and β –haematin inhibition (10,13,14]. Following similar studies to those published previously, the association constant of complexes 1–6 with ferriprotoporphyrin IX (Fe(III)PPIX) were determined (Table 2). This association was followed by spectrophotometric titration at the 402 nm Soret band in aqueous DMSO at pH 5, to the equation for a 1:1 complexation model using nonlinear least squares fitting, strictly following the procedure of Egan et al. [21]. As an example, in Figure 2 is shown the needed concentration of complex 6 to reach the saturation point, and the hypochromism was approximately 70%. The log K value of 5.01 ± 0.01 obtained for CQDP under the present experimental conditions (pH 7.5) is in excellent agreement with the value reports for this method [21], in the case of log K for the studied complexes (1–6) was obtained fitted in a strictly analogous manner, the range of log K values are between 4.05 and 4.87 (Table 2), this indicates that complexes 1–6 interact with haematin comparably that CQDP does, under used conditions. Additionally, it is important of mentioning that these values are in the range of ferroquine, which has demonstrated high anti-malarial activity [5].

A first approximation to study the inhibition of β-haematin formation was done qualitatively using FTIR spectroscopy, monitoring the absence of characteristic β-haematin bands at 1660 and 1210 cm^−1^ [22] which are obtained in the control experiment in the absence of CQ and studied metal complexes. However, the IR spectra obtained when the same experiment was done in the presence of 3 equivalents of CQ and each studied metal complexes (separate experiments) showed the absence of the bands at 1660 and 1210 cm^−1^ making evident that β-haematin was not produced, similarly to previous observations for CQ, other organic antiplasmodial compounds [22] and metal-CQ complexes [5].

The interesting results previously discussed motivated the study of the effect of complexes 1–6 on the haem aggregation inhibition activity assays (HAIA), which was determined using two set of experiments, the first one was carried out in buffer while a second set was performed in an interface n-octanol/ aqueous buffer interface; these results were compared to those ones obtained for CQDP as control (Table 2).

The IC_50_ values measured for these complexes in acetate buffer at pH 5 shown that complexes 1–6 inhibit the haem aggregation process at higher IC_50_ than the one obtained for CQDP. Similar results were obtained by this method for [RuCl_2_(CQ)]_2_ [13] and [Au(CQ)(PPh_3_)]PF_6_ [14] complexes previously published, where no correlation was observed between the inhibition of the haem aggregation in buffer with their anti-malarial activities against CQR strains. Therefore, a more realistic condition in the interfaces where the β-haematin assembles rapidly and spontaneously occurred was used following an adaptation of the procedure described by Egan *et al.* [24] and reported by Sánchez-Delgado *et al.* [25], in which the haematin is carefully introduced close to the interface after the drug has been equilibrated between the two phases, the overall aggregation process is much faster (60 min) through this method. The activity trend changes drastically with respect to the results of the assay in aqueous buffer. The complexes 1–6 was comparable or 2.3 times better inhibitor than CQDP on the inhibition of the haem aggregation near the interface, providing a plausible explanation for the enhanced anti-plasmodial activity displayed for these studied metal complexes.

## Conclusion

The synthesis and characterization of a new Pd-CQDP (6) complex was achieved and together with complexes 1–5 were tested against two strain of malarial parasite, finding that all these complexes displayed very high anti-malarial activity against the CQS strain (3D7), while only complexes 2, 3 and 4 were up to four times more active than CQDP against CQR strain (W2). Complexes 1–6 interact with haem and inhibit β-haematin formation to a greater extent than chloroquine diphosphate (CQDP) and other known metal-based anti-malarial agents. Putting together all of these results, it is possible to suggest that the enhanced of the antiplasmodial activity displayed for these studied metal complexes is related to their ability to inhibit β-haematin formation.

## Electronic supplementary material

Additional file 1: **Spectroscopic and analytical characterization of complex Pd(CQDP)**_**2**_ **I**_**2**_ **(6).** (DOCX 14 KB)
