# Supplementary material for: Metal-chloroquine derivatives as possible anti-malarial drugs: evaluation of anti-malarial activity and mode of action
Source: Malar J. 2014 Dec 3;13:471. doi: 10.1186/1475-2875-13-471 (PMC4289335; doi:10.1186/1475-2875-13-471)
Supplement: Supplementary file 1 — Additional file 1: Spectroscopic and analytical characterization of complex Pd(CQDP) 2 I 2 (6). (DOCX 14 KB) [file 12936_2014_3626_MOESM1_ESM.docx]

Additional data 1 Spectroscopic and analytical characterization of complex Pd(CQDP)_2_I_2_(**6**)

Yield 95%; Elemental analysis (%) Calc. for C_36_H_64_N_6_Cl_2_I_2_O_16_P_4_Pd (1389.98 g.mol^-1^): C 31.1; N 6.0; H 4.6. Found: C 31.4; N 5.9; H 4.8. ESI-MS (MeOH): (M- 4H_3_PO_4_^-^) 1000.03; IR: υ (N-H) 3332 cm^-1^; υ (C=C) 1612 cm^-1^; υ (C=N) 1583 cm^-1^. UV-vis (DMSO) 258, 330 and 344 nm. ε (DMSO) [(λ nm)]: 35400 M^-1^cm^-1^ (258 nm), and 36200 M^-1^cm^-1^ (344 nm). ^1^H-NMR (DMSO-d_6_; δ ppm): 13.65 (1H; s, NH^+^); 8.86 (2H; NH^+^ y NH); 8.64 (1H; d; J=9.15; H5); 8.58 (1H; d; J=7.14; H2); 7.91 (1H; d; J=2.35; H8); 7.82 (1H; dd; J^1^, 2.02 Hz and J^2^, 9.06 Hz; H6); 6.99 (1H; d; J=7.20; H3); 4.16 (1H; m; H1’); 3.10 (6H; m; H4’ and H5’); 1.72 (4H; m; H2’ and H3’); 1.31 (3H; d; J, 6.24 Hz; H1´´); 1.16 (6H, t, H6´);^13^C-NMR (DMSO-d_6_; δ ppm): 155.38 (C9); 143.62 (C2); 138,99 (C4); 138.66 (C7); 127.30 (C6); 126.25 (C5), 119.59 (C8); 115.85 (C10); 99.31 (C3); 51.09 (C4’); 49.67 (C1’); 46.93 (C5’); 32.44 (C2’); 20.62 (C3’); 20.00 (C1’’); 9.11 (C6’).^31^P-NMR (DMSO-d6; δ ppm): -0.07 (H_2_PO_4_^-^). Molar conductivity in Dimethylformamide (DMF), ΛM = 305 ± 9 ohm^-1^ cm^2^ mol^-1^.
